# HIV self-testing: finding its way in the prevention tool box

**DOI:** 10.1186/s12916-020-01852-y

**Published:** 2020-11-30

**Authors:** Katrina F. Ortblad, Joanne D. Stekler

**Affiliations:** 1 Department of Global Health, University of Washington, 908 Jefferson St, Seattle, WA 98104 USA; 2 Department of Medicine, University of Washington, Seattle, USA; 3 Department of Epidemiology, University of Washington, Seattle, USA

## Background

One in five people living with HIV globally remains unaware of their HIV status [1]. There has been much emphasis on identifying these individuals so they can link to HIV treatment services and modify behaviors that contribute to HIV transmission [1]. Novel intervention strategies have been developed to expand access and reach of HIV testing services, including assisted partner notification systems, community-based testing, and more recently HIV self-testing (HIVST) [2]. HIVST, which has some advantages (e.g., increased privacy and convenience) and disadvantages (e.g., lower sensitivity of oral fluid self-tests and potential challenges with linkage to care) over these other strategies, has gained particular enthusiasm and is currently being scaled in a number of high HIV prevalence settings and populations [3].

HIVST may be especially well-suited for key populations, including men who have sex with men (MSM) and female sex workers (FSWs), by addressing additional barriers these populations face to facility-based testing (e.g., stigma, discrimination, and inconvenient clinic hours). In this study, Dr. Witzel and colleagues conducted a systematic review and meta-analysis to understand the effect of HIVST versus standard facility-based HIV testing for key populations [4]. Overall, the authors found that HIVST increased testing uptake, frequency, and yield of positive results for cisgender MSM and transgender persons but not for FSWs. Of note is that the increased yield of positive results for MSM and trans people was due to a mix of increased diagnosis of established infections and, in at least one study, a higher number of incident cases among those randomized to HIVST. In terms of harms, HIVST did not impact STI testing, condom use, or social harm for all key populations but was associated with lower reported linkage to HIV treatment across populations. The authors conclude that HIVST is safe for key populations but strategies to improve linkage to treatment are needed for effective roll-out.

## HIVST as a gateway to HIV/STI prevention

One role for HIVST not included in the review is its potential to improve linkage to HIV prevention interventions. The majority of individuals that self-test (even in high incidence populations) will test negative for HIV infection. These individuals should not be forgotten; rather, HIVST delivery should be paired with resources to increase engagement in and access to behavioral (e.g., condom distribution and negotiation skills) and biomedical (e.g., pre-exposure prophylaxis [PrEP]) HIV prevention interventions, as well as uptake of testing for sexually transmitted infections (STI) and other comorbid conditions. This may be especially important for key populations who often are pessimistic about their ability to remain HIV uninfected and have very high rates of STI incidence because of the activities they engage in, such as selling condomless sex for economic survival. Newly acquired knowledge of HIV-negative status alone can be a powerful intervention; as demonstrated among Ugandan and Zambian FSWs who significantly increased condom use with clients when they learned they were HIV uninfected [5]. Additionally, using HIVST as a catalyst for engagement in HIV prevention and STI diagnosis interventions might result in the greatest benefits and smallest risks. Because oral fluid self-tests are significantly less sensitive than other tests during early HIV infection, some mathematical models caution that replacement of blood-based facility HIV testing with oral-fluid self-testing may increase HIV prevalence among MSM [6].

## HIVST to support overburdened health systems

HIVST also has the potential to support health systems, saving both time and costs for patients and providers. For example, HIVST could be used to reduce or eliminate engagement with health facilities for interventions that require regular HIV testing (e.g., PrEP). HIVST-supported models of care delivery outside health facilities have recently been used as a risk mitigation strategy during the COVID-19 pandemic in the USA. Similar models are currently being tested in Kenya, including a trial comparing biannual PrEP facility visits with interim HIVST vs. standard quarterly PrEP facility visits (ClinicalTrials.gov: NCT03593629) and a pilot study of pharmacy-based PrEP delivery with provider-assisted HIVST (ClinicalTrials.gov: NCT04558554). HIVST could additionally improve flow and efficiencies within health facilities. For example, HIVST could enable patients to test for HIV while waiting to meet with providers at health facilities, potentially reducing time spent with providers or freeing time with provider to discuss other concerns that may improve quality of care and patient satisfaction [7]. Key populations may especially benefit from HIVST-supported interventions that reduced engagement with health facilities, which are often difficult for these populations to access, or free time with providers to discuss unique life circumstances and get referral to mental health or family planning services.

## HIVST to increase self-efficacy and support mental health outcomes

Evidence suggests that access to HIVST can have mental health benefits. As demonstrated in a recent study among FSWs that applied Kabeer’s empowerment framework [8], access to HIVST can increase feelings of empowerment by increasing time and money (resources), control of testing circumstances and status disclose (agency), and a sense of competency (achievements). Additionally, HIVST can increase individuals’ control of sexual engagements, by availing a way to HIV test potential sexual partners (i.e., point-of-sex testing). In another study, FSWs reported that even if a client refused HIVST, that would give them an indication of the client’s likely HIV status, which would influence their sexual decision making [9]. This, however, comes with potential challenges associated with the low sensitivity of oral-fluid HIVST during early infection, resulting in false-negative test results and false perceptions of sexual safety. Recent knowledge of HIV status (be it positive or negative), acquired through HIVST, may also decrease depressive symptoms among key populations that often carry the constant stress of potential HIV infection [10].

## Conclusions

The potential of HIVST should not be limited to yielding positive results and facilitating linkage to treatment. While this should remain an important goal of HIVST programs, we should also capitalize on the unique characteristics of this testing technology (e.g., privacy, mobility) and utilize HIVST in ways that facilitate linkage to HIV prevention interventions, support health systems, and improve mental health outcomes. Additional work needs to be done to develop and test novel HIVST-supported interventions that can help us maximize the potential of this new testing technology.

## Data Availability

Not applicable
